# Apatinib induces endoplasmic reticulum stress-mediated apoptosis and autophagy and potentiates cell sensitivity to paclitaxel via the IRE-1α–AKT–mTOR pathway in esophageal squamous cell carcinoma

**DOI:** 10.1186/s13578-021-00640-2

**Published:** 2021-07-06

**Authors:** Yu-Ming Wang, Xin Xu, Jian Tang, Zhi-Yong Sun, Yu-Jie Fu, Xiao-Jing Zhao, Xiu-Mei Ma, Qing Ye

**Affiliations:** 1 Department of Thoracic Surgery, Renji Hospital, School of Medicine, Shanghai Jiao Tong University, Shanghai, 200127 People’s Republic of China; 2 Department of Radiation Oncology, Renji Hospital, School of Medicine, Shanghai Jiao Tong University, Shanghai, 200127 People’s Republic of China

**Keywords:** Apatinib, ER stress, Autophagy, Apoptosis, Paclitaxel, Esophageal squamous cell carcinoma

## Abstract

**Background:**

Apatinib, a novel vascular endothelial growth factor receptor-2 (VEGFR-2) tyrosine kinase inhibitor, has been approved for the treatment of metastatic gastric cancer and other tumors. Apatinib exerts antiproliferative and proapoptotic effects in different kinds of cancer cells. However, the molecular mechanisms by which apatinib effective against esophageal squamous cell carcinoma (ESCC) have only been partially researched and whether it has a sensitizing effect on paclitaxel remains unclear.

**Materials and methods:**

The effects of apatinib or paclitaxel on endoplasmic reticulum (ER) stress, autophagy, apoptosis and proliferation of ESCC cell lines were evaluated. Western blot and immunohistochemistry analyses were performed to detect the expression of related genes. The weight and volume of xenograft tumors in mice were measured.

**Results:**

In the current study, we elucidated the antiproliferative and ER-stress-mediated autophagy-inducing effects of apatinib on ECA-109 and KYSE-150 esophageal squamous cancer cells and identified the underlying mechanisms of its action. We demonstrated that apatinib not only inhibited the proliferation and induced the apoptosis of ESCC cells, but also activated ER stress and triggered protective autophagy. Moreover, inhibiting autophagy by chloroquine (CQ) enhanced the apatinib-induced apoptosis of ESCC cells through the IRE-1α–AKT–mTOR pathway. In addition, we showed, for the first time, the paclitaxel combined with apatinib and CQ exhibited the best antitumor effect on ESCC both in vivo and in vitro via the IRE-1α–AKT–mTOR pathway.

**Conclusions:**

Our data showed that apatinib induced ER stress, autophagy and apoptosis in ESCC. Inhibiting autophagy by CQ enhanced apatinib-induced apoptosis. The combination of apatinib and CQ sensitized ESCC cells to paclitaxel to induce apoptosis through the IRE-1α–AKT–mTOR signaling pathway, thus providing the basis for its use in innovative anticancer therapeutic strategies.

**Graphic abstract:**

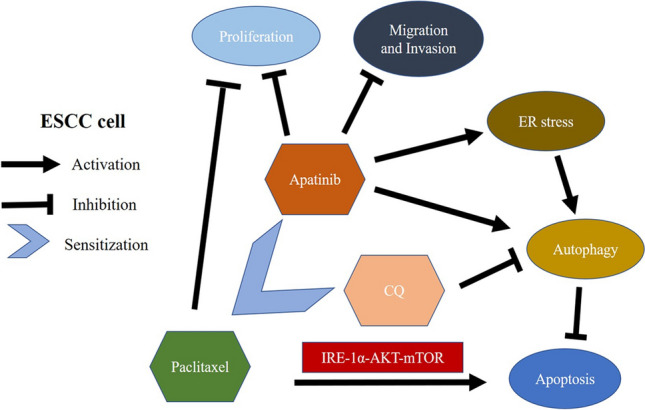

**Supplementary Information:**

The online version contains supplementary material available at 10.1186/s13578-021-00640-2.

## Introduction

ESCC is the type of esophageal cancer predominant in East Asia, especially in China, where it accounts for more than 90% of cases [1, 2]. It has been reported that there are more than 280,000 new cases of ESCC every year in China [3–6]. Despite advances in diagnosis and therapy in recent decades, the 5-year survival rate remains low at an estimated 15–20% [7].

Surgery is the primary treatment for early-stage ESCC; however, more than one-half of patients are found to have locally advanced cancer at diagnosis, and therefore, they are offered neoadjuvant or adjuvant therapy. The combination of platinum-based 5-fluorouracil and paclitaxel is currently the most frequently used perioperative chemotherapy [8]. The combination of chemotherapy and targeted therapy has provided opportunities for improving outcomes in recent years. However, only 3 targeted therapeutic agents including trastuzumab, ramucirumab, and pembrolizumab, have been approved by the FDA for use in esophageal and EGJ cancers [9]. Most researches about targeted therapy are still in the clinical or basic research stage. Apatinib, as a novel VEGFR-2 tyrosine kinase inhibitor [10, 11], is the second antiangiogenic drug that has been approved for the therapy of advanced metastatic gastric cancer [12]. In addition to gastric cancer, apatinib also plays an important role in mitigating other solid tumors, including thyroid cancer, gynecologic cancer, hepatocellular carcinoma (HCC) and esophageal cancer [13–16]. There are relatively few studies of apatinib with respect to esophageal cancer, and the application value and corresponding mechanism of apatinib when combined with chemotherapy are not sufficiently clear [13, 17–19].

The endoplasmic reticulum is a dynamic organelle with significant functions including folding of newly synthesized proteins and maintenance of calcium homeostasis [20, 21]. ER stress occurs when homeostatic processes are disrupted and results in the accumulation of unfolded or misfolded proteins [22]. To resist ER stress and maintain cellular homeostasis, autophagy is triggered to degrade and recycle cytoplasmic materials and thus has mostly a pro-survival function in cancer cells. Moreover, autophagy and apoptosis are closely related. Generally, autophagy prevents the induction of apoptosis through the inactivation of caspases. However, under special circumstances, autophagy or autophagy-related proteins might facilitate apoptosis induction. These findings indicate that the duality of autophagy might be related to the regulation of apoptosis of cells under ER stress [23, 24].

In this study, we aimed to clarify the potential therapeutic effects and relevant molecular mechanisms of apatinib in ESCC in vivo and in vitro and to explore the sensitizing effect of apatinib on cells treated with paclitaxel. We first found that apatinib had an effective antitumor effect by inhibiting tumor proliferation, invasion and migration in ESCC. Then, we found that apatinib induced ER stress, autophagy and apoptosis. Inhibiting autophagy by CQ administration significantly enhanced anticancer effects of apatinib via the IRE-1α–AKT–mTOR signaling pathway. Finally, we explored whether the combination of apatinib and CQ could sensitize ESCC cells to paclitaxel to induce apoptosis both in vivo and in vitro.

## Materials and methods

### Cell cultures

ECA-109 cells (RRID: CVCL_6898) and KYSE-150 cells (RRID: CVCL_1348) (Stem Cell Bank, Shanghai, China) were authenticated using STR (or SNP) profiling within the last three years and were cultured in RPMI-1640 or DMEM (Sigma, St. Louis, MO, USA) with 10% FBS (Gibco, USA), 100 U/ml streptomycin and 100 U/ml penicillin (Millipore, TMS-AB2-C) at 37 °C in a humidified incubator with 5% CO_2_. All experiments were performed with mycoplasma-free cells. To detect autophagic flux, the ECA-109 and KYSE-150 cells were transfected with RFP-GFP-hLC3 lentivirus (Jiman, Shanghai); the specific procedures were described previously [25].

### Reagents and antibodies

Apatinib was obtained from Hengrui Medicine Co., Ltd. (Jiangsu, China). Paclitaxel, CQ, SC79, rapamycin and bafilomycin A1 were purchased from Selleck Chemicals (Houston, TX, USA). Paclitaxel was purchased at a concentration of 10 mM, which was soluble in DMSO. Serum-free medium was used to dilute the drug to working concentration (10 μM) and the v/v% was 0.1% (1:1000). Apatinib was purchased in the form of powder. 10 mg apatinib was dissolved in 25.159 μl DMSO to get the drug with a concentration of 1 M. Then, the serum-free medium was used to dilute the drug to working concentration (25 mM) and the v/v% was 2.5% (1:40).

Antibodies including anti-Bax (89477), anti-Bcl2 (15071), anti-Caspase3 (14220), anti-Cleaved-Caspase3 (9664), anti-PARP (9532), anti-Cleaved-PARP (5625), anti-Chop (2895), anti-GRP78 (3177), anti-IRE-1α (3294), anti-JNK (9252), anti-Beclin1 (3495), anti-LC3 (12741), anti-P62 (23214), anti-AKT (4685), anti-pAKT (4060), anti-mTOR (2983), anti-pmTOR (5536), anti-GAPDH (2118) were all purchased from Cell Signaling Technology (Beverly, MA, USA).

### Cell viability assays

A Cell Counting Kit-8 (Dojindo, Kumamoto, Japan) was used to assess the cytotoxicity of drugs on ESCC cell lines. Approximately 2500 cells were plated in 96-well plates 24 h before they were treated with different drugs. The absorbance at 450 nm was detected by a microplate reader.

### Colony formation assay

A total of 1000 cells were seeded in 6-well plates and incubated for 2 weeks after treatment with drugs and then stained with 1% crystal violet before being photographed and quantified.

### Apoptosis rate and cell cycle analyses

Cells were seeded in six-well plates for 24 h and then treated with apatinib or paclitaxel for another 24 h. The harvested cells were washed twice with cold PBS and treated with 5 μl of propidium iodide (PI, 50 μg/ml) and 3 μl of FITC-Annexin V for 15 min at room temperature in the dark. Three hundred microlitres of 1xbinding buffer were added to each sample. The number of apoptotic cells was determined with a FACS-Caliber system (BD Biosciences, USA). For cell cycle analysis, the preparation of cells was similar to that described for the apoptosis analysis and were fixed overnight in 70% ethanol at 4 °C. Then, the cells were washed twice with cold PBS and incubated with PI (50 μg/ml) for one-half hour in the dark. Finally, all samples were analyzed by a FACS Caliber system. In the above experiment, 3 independent events were set for each group, and the results were averaged.

### Western blot analysis

Protein samples from treated cells were extracted and quantified by a BCA protein assay kit (Pierce, Rockford, USA), subjected to SDS-PAGE and transferred to PVDF membranes (Millipore, Temecula, California, USA). Then, the bolts were probed with primary and secondary antibodies.

### Confocal microscopy

We used 4% paraformaldehyde (Sigma) to fix cells for 20 min and 0.1% Triton X-100 (Sigma) to permeabilize cells for 15 min at room temperature. Then, the cells were washed with PBS and blocked with PBS containing 0.5% bovine serum albumin (BSA) and 0.15% glycine (BSA buffer) for 1 h at room temperature. Finally, the slices were treated with DAPI (Sigma) and imaged by confocal microscopy (Zeiss).

### ***Intracellular Ca***^***2***+^***detection***

We used Fluo3 AM (S1056, Beyotime, Shanghai, China) to detect intracellular Ca^2+^ levels. Flow cytometry was used to detect the fluorescence intensities of Fluo3 signals and determine Ca^2+^ binding.

### Electron microscopy

After treatment, cells were fixed in 2% glutaraldehyde and 2% paraformaldehyde in 0.1 mol/l sodium phosphate buffer (pH 7.4) at 4 °C for 3 h. After postfixing in 1% osmium tetroxide in the same buffer for 2 h and gradual dehydrated in an alcohol gradient, the samples were embedded in a mixture of EPON 618 and epoxypropane. Ultrathin sections were stained with 5% uranyl acetate and Reynold’s lead citrate and semithin sections were stained with toluidine blue. An electron microscope equipped with a digital camera was used to examine the sections.

### RNA interference

Cells were treated with IRE-1α or Beclin1 siRNA (200 nM, BioeGene, Shanghai, China). The siRNA sequences of IRE-1α were as follows: siRNA-1 5ʹ-CCAAGAUGCUGGAGAGAUUTT-3ʹ (sense); 5ʹ-AAUCUCUCCAGCAUCUUGGTA-3ʹ (antisense). siRNA-2 5ʹ-GGAAGUUAUCAACCUGGUUTT-3ʹ (sense); 5ʹ-AACCAGGUUGAUAACUUCCTG-3ʹ (antisense). siRNA-3 5ʹ-GCAGAAGGACUUUGCGCAUTT-3ʹ (sense); 5ʹ-AUGCGCAAAGUCCUUCUGCTC-3ʹ (antisense). The siRNA sequences of Beclin1 were as follows: siRNA-1 5ʹ-GCUCAGUAUCAGAGAGAAUTT-3ʹ (sense); 5ʹ-AUUCUCUCUGAUACUGAGCTT-3ʹ (antisense). siRNA-2 5ʹ-GGUCUAAGACGUCCAACAATT-3ʹ (sense); 5ʹ-UUGUUGGACGUCUUAGACCCT-3ʹ (antisense). siRNA-3 5ʹ-CCCAGGAGGAAGAGACUAATT-3ʹ (sense); 5ʹ-UUAGUCUCUUCCUCCUGGGTC-3ʹ (antisense). Western blotting was used to detect the effect of IRE-1α or Beclin1 siRNAs, and select the siRNA with the best knockout effect for follow-up experiments (Additional file 1: Figure S1a–f).

### Xenograft tumor model, immunochemistry and TUNEL assay

We purchased four-week male BALB/c nude mice from the Institute of Zoology, Chinese Academy of Sciences of Shanghai and carried out all the experiments strictly according to the Guide for the Care and Use of Laboratory Animals [26]. Research was approved by the Institutional Animal Care and Use Committee at the Shanghai Jiaotong University School of Medicine (Approval ID: A-2018-024). ECA-109 cells (1 × 10^6^ cells in 100 μl of PBS per mouse) were subcutaneously injected into the right flanks of the nude mice by. After 14 days, all animals were randomly assigned to 6 groups, which were shown in Table 1. Tumor volume was calculated on the basis of weekly measurement using the following formula: V = 0.5 × length x width^2^. All mice were sacrificed 6 weeks after ECA-109 cell inoculation, and xenograft tumors were weighed and fixed for immunohistochemistry (IHC) staining. A TUNEL assay was carried out to determine the apoptotic cells using an in situ cell death detection kit with Fluorescein (Roche Applied Science, USA).

**Table 1:** Animal experimental groups

Name of groups	Numbers of animals	Drug intervention
Control group	4	Use PBS only
Apatinib group	4	60 mg/kg, daily oral gavage
CQ group	4	60 mg/kg, daily oral gavage
Apatinib + CQ group	4	60 mg/kg, daily oral gavage of both drugs
Paclitaxel group	4	15 mg/kg, twice a week, intraperitoneal injection
Apatinib + CQ + Paclitaxel group	4	Apatinib and CQ: 60 mg/kg, daily oral gavage; Paclitaxel: 15 mg/kg, twice a week, intraperitoneal injection

### Statistical analyses

All data were analyzed using GraphPad 6.0 and SPSS 18.0 software. The data were expressed as the means ± standard deviation. For continuous variables, Student’s t-test and ANOVA were used to determine the significance of differences among groups. Mann–Whitney U-test was used for comparing tumor volumes. For categorical variables, Pearson’s chi-square test, continuity correction, or Fisher's exact or likelihood ratio were used. A p-value less than 0.05 was considered to be statistically significant.

## Results

### Apatinib inhibited ESCC cell proliferation, migration and invasion

**Fig. 1:**
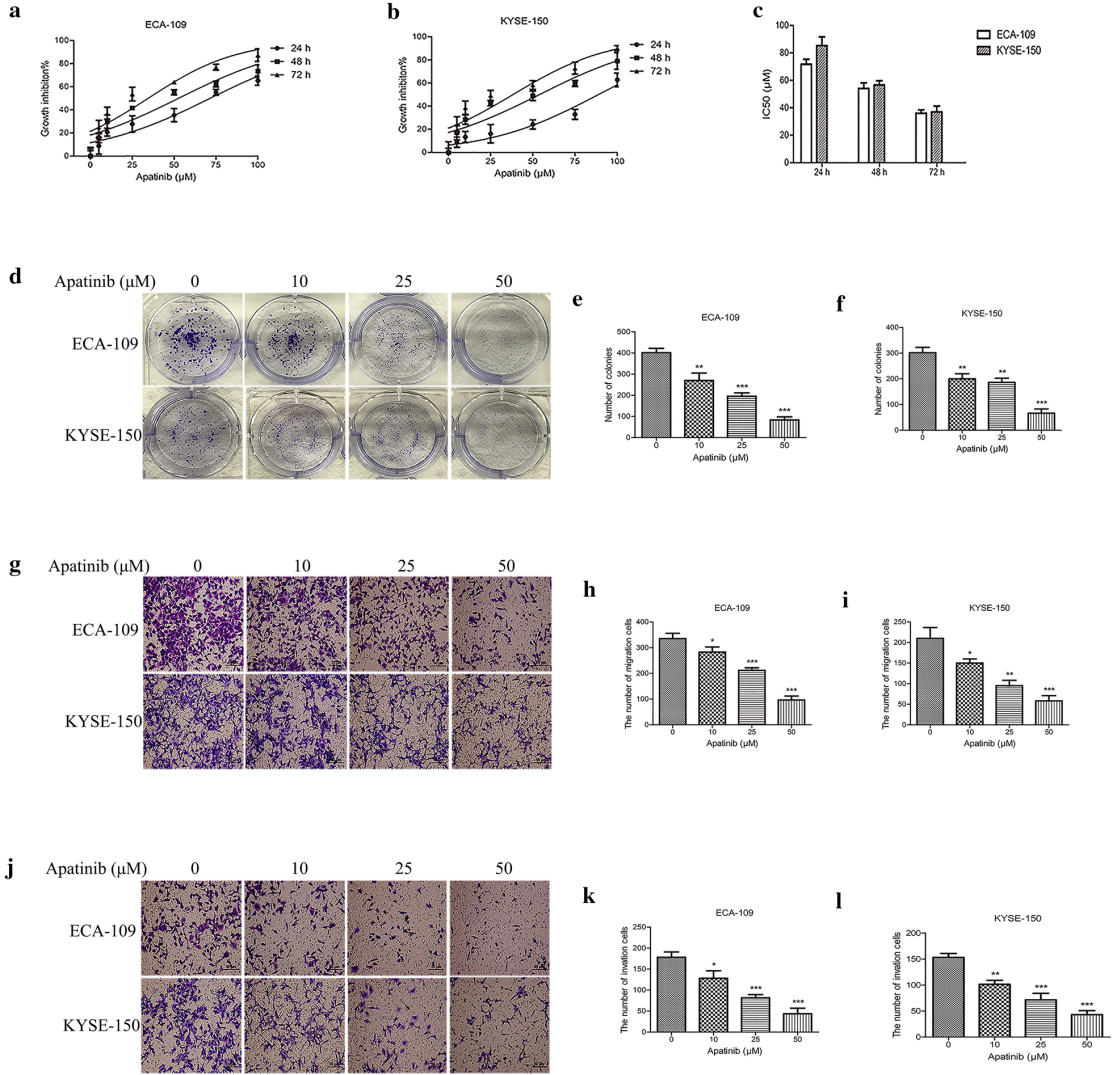
Apatinib inhibited the proliferation, migration and invasion of ESCC cells. **a**, **b** Cell viability was evaluated by CCK8 assay. **c** IC50 values were calculated. **d**–**f** Representative images and quantification of clone formation experiments were shown. **g**–**i** Apatinib inhibited the migration of ESCC cells. **j**–**l** Apatinib inhibited the invasion of ESCC cells. * p < 0.05, ** p < 0.01, *** p < 0.001 compared with control group

To explore the effect of apatinib on the proliferation of ESCC cells, we treated the cells with 7 different apatinib concentrations and evaluated the cells at 3 different time points. The results showed that apatinib significantly inhibited the proliferation of ESCC cells (Fig. 1a, b). IC50 values were calculated, and the results were presented in Fig. 1c. Next, plate clone formation experiments were conducted to verify our conclusions. The results showed that apatinib significantly inhibited colony formation (Fig. 1d–f). In addition, the effect of apatinib on the invasion and migration of ESCC cells was explored. The results showed that apatinib significantly inhibited the invasion and migration ability of tumor cells in a dose-dependent manner (Fig. 1g–l). In summary, apatinib inhibited the proliferation, invasion and migration of ESCC cells.

### Apatinib arrested the cell cycle and induced apoptosis

**Fig. 2:**
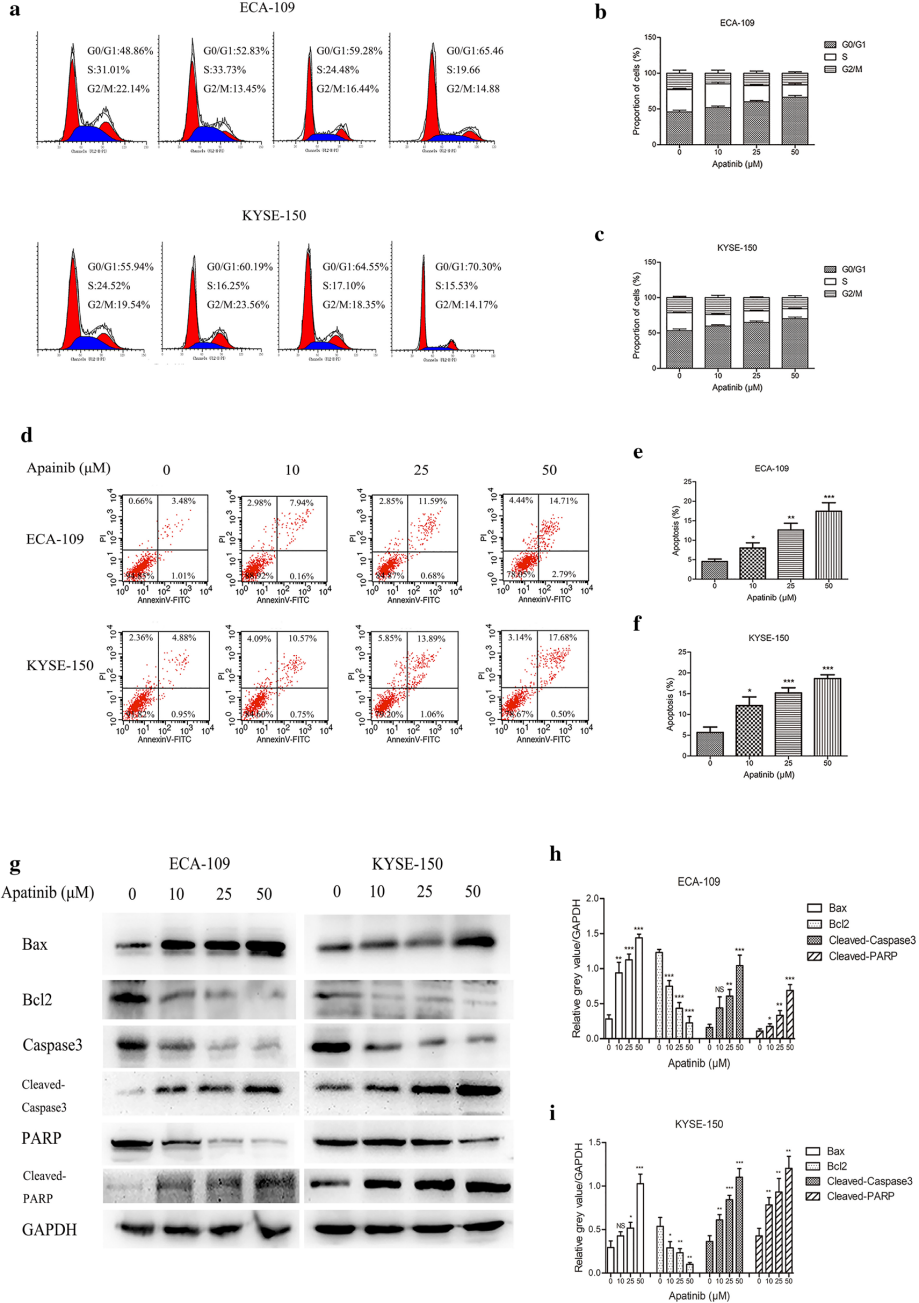
Apatinib induced cell cycle arrest and the apoptosis of ESCC cells. **a**–**c** Cell cycle phase distribution was measured by flow cytometry. **d**–**f** Annexin V-FITC and propidium iodide (PI) staining were used to measure apoptotic ESCC cells treated with apatinib for 24 h. **g**–**i** Western blot analysis was performed to measure the expression levels of apoptosis markers after cells were incubated with apatinib for 24 h. * p < 0.05, ** p < 0.01, *** p < 0.001 compared with control group

Then, we tried to elucidate the mechanism by which apatinib inhibited the proliferation of ESCC cells. We firstly used flow cytometry to study the effect of apatinib on the cell cycle. The results indicated an evident decrease in cell numbers in the S phase, while the number of cells in the G0/G1 phase markedly increased after treatment with apatinib (ECA-109 cells 59.89 ± 1.61 (apatinib 25 μM) vs 45.83 ± 1.95 (control), p < 0.01, 66.39 ± 2.03 (apatinib 50 μM) vs 45.83 ± 1.95 (control), p < 0.001; KYSE-150 64.98 ± 1.58 (apatinib 25 μM) vs 53.21 ± 1.98 (control), p < 0.01, 70.20 ± 1.84 (apatinib 50 μM) vs 53.21 ± 1.98 (control), p < 0.001) (Fig. 2a–c). These results showed that apatinib inhibited the G1-S transition of ESCC cells, thereby inhibiting cell proliferation. We secondly explored the effects of apatinib on the apoptosis of ESCC cells. Flow cytometry and western blotting were used to evaluate the apoptosis rate. The results of flow cytometry revealed that apatinib significantly increased the proportion of apoptotic cells compared with the control group (Fig. 2d–f). The western blot results showed that the expression levels of Bax, Cleaved-Caspase3, and Cleaved-PARP were significantly increased, while the expression levels of Bcl2, Caspase3, and PARP levels were decreased after treatment with apatinib (Fig. 2g–i).

### Apatinib induced ER stress and autophagosome formation

**Fig. 3:**
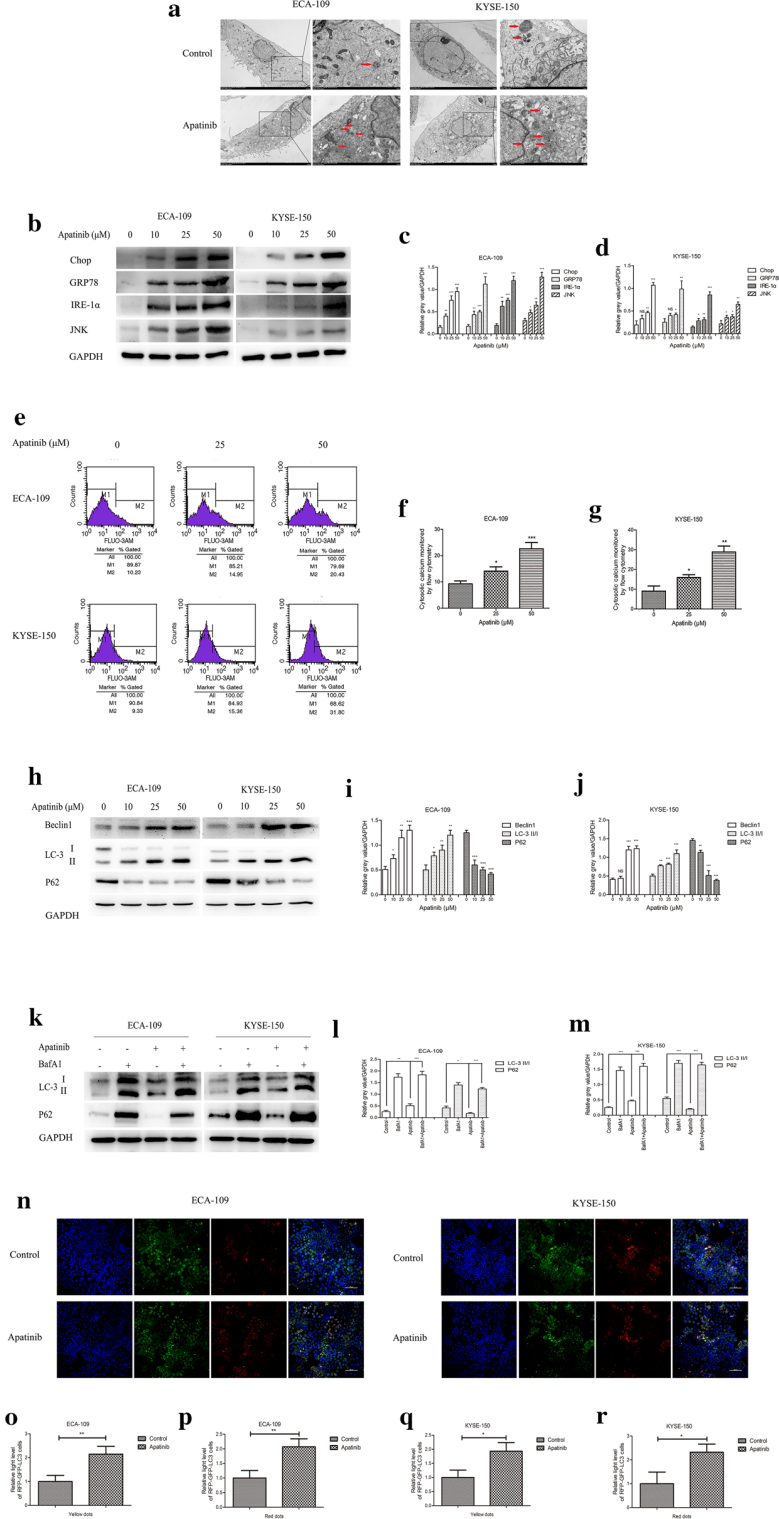
Apatinib induced ER stress and autophagy in ESCC cells. **a** Electron microscopy was used to view changes in the rough endoplasmic reticulum, formation of vacuolization and the number of autophagosomes and/or autolysosomes. *N* nucleus, *M* mitochondria, *RER* rough endoplasmic reticulum, *ASS* autolysosome, *GO* Golgi apparatus, *LD* lipid droplet. **b**–**d** Western blot analysis was performed to measure the expression levels of ER stress markers after cells were incubated with apatinib for 24 h. **e**–**g** The level of cytosolic calcium was evaluated by flow cytometry. **h**–**j** The expression levels of autophagy markers in cells incubated with apatinib for 24 h were measured by western blotting. **k**–**m** The expression levels of LC-3, P62, and GAPDH in cells treated with 25 μM apatinib for 24 h or 50 nM BafA1 for 12 h were measured by western blotting. **n**–**r** Representative images and quantification of autophagosomes shown as yellow dots and autolysosomes shown as red dots were shown in cells transfected with RFP-GFP-LC3 lentivirus and incubated with 25 μM apatinib for 24 h. * p < 0.05, ** p < 0.01, *** p < 0.001 compared with control group

Apoptosis has been reported to be closely related to ER stress and autophagy [23]. Therefore, we focused on the effect of apatinib on ER stress and autophagy in ESCC. Figure 3a showed that compared with the control group, the rough ER in the apatinib group was expanded, the surface ribosomes were partially shed, the Golgi apparatus showed severe hypertrophy, and more autophagic lysosomes were formed in the cytoplasm, indicating that both ER stress and autophagy had been activated. Then, we verified the occurrence of the two phenomena. For assessing ER stress, western blot analysis was performed to detect UPR-targeting markers including Chop, GRP78, IRE-1α and JNK. The results showed that the expression of these markers was significantly up-regulated after treatment with apatinib (Fig. 3b–d). Maintenance of calcium homeostasis is one of the crucial functions of the endoplasmic reticulum. When ER stress occurs, the calcium ion balance is obviously disordered. Therefore, we further evaluated the level of calcium in the cytoplasm. Fluo3AM was used to label calcium ions, which were subsequently detected by flow cytometry, and the results showed that the level of calcium ions significantly increased after cell treatment with apatinib (Fig. 3e–g). For assessing autophagy, we first performed a western blot analysis to detect the expression levels of classic markers, such as LC3, Beclin1, and P62, and the results were shown in Fig. 3h–j. The addition of apatinib significantly upregulated the expression levels of LC3 II and Beclin1 and inhibited P62 expression, compared with the control group. However, since autophagy was a dynamic process, we were not sure whether apatinib activated early autophagy or suppressed late autophagy; therefore, BafA1 was used for further exploration. Figure 3k–m showed that the upregulation effect of apatinib on LC3 expression was more obvious when apatinib was combined with BafA1. Then, the RFP-GFP-LC3 fusion gene lentivirus was used to transfect ESCC cells. In the analysis, yellow dots represented autophagosomes and red dots represented autolysosomes. The results showed that the number of yellow and red dots increased significantly after treatment with apatinib, suggesting that apatinib significantly increased autophagic flux (Fig. 3n–r). These results indicated that ER stress and autophagy were activated by apatinib in the ESCC cells.

**Fig. 4:**
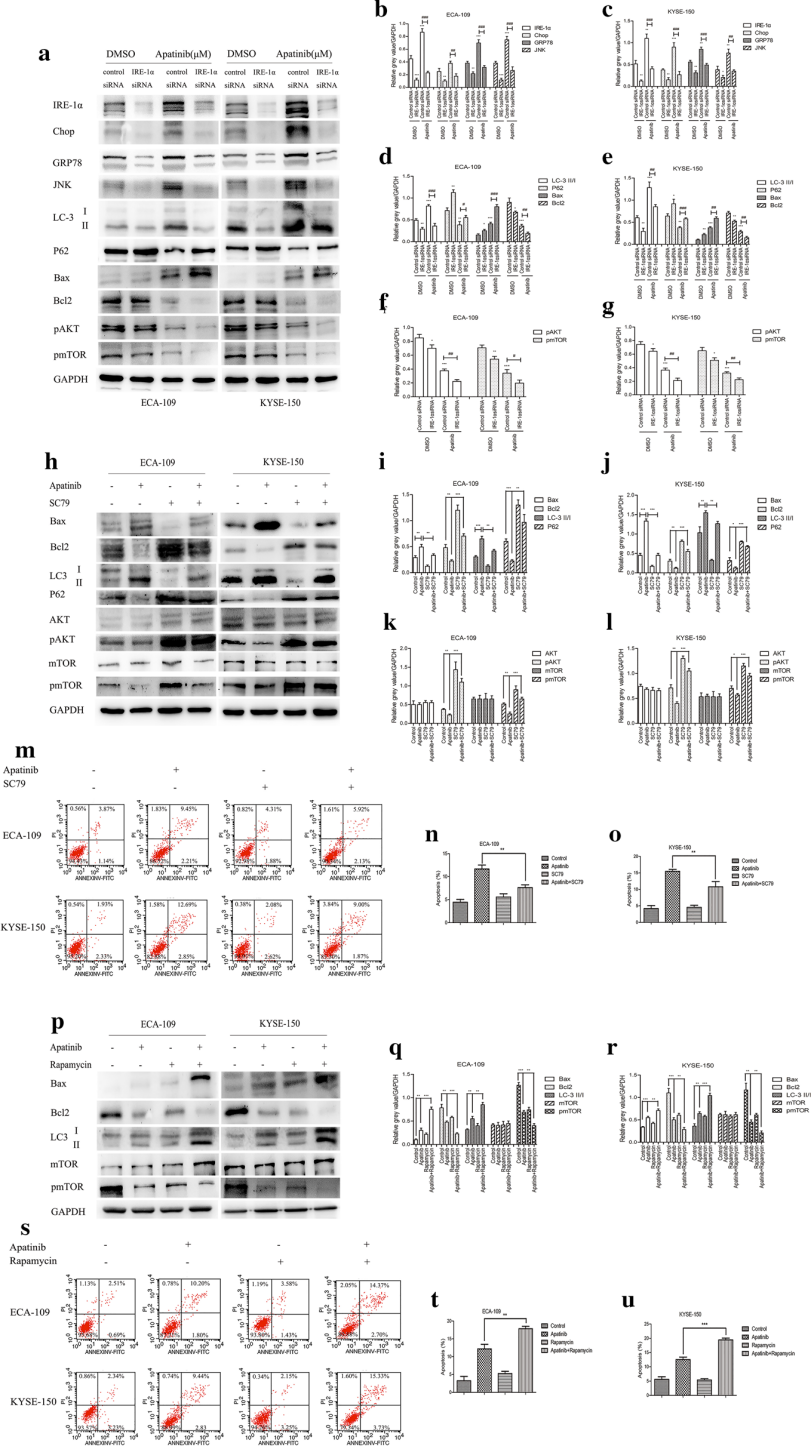
Apatinib affected autophagy and apoptosis via the IRE-1α–AKT–mTOR pathway. **a**–**g** ECA-109 and KYSE-150 cells were treated with 25 μM apatinib or DMSO for 24 h after transfection with control siRNA or IRE-1α siRNA. The expression levels of related genes were measured by western blotting. **h**–**o** Cells were treated with apatinib (25 μM) with or without SC79 (4 μg/ml). The expression levels of related genes were measured by western blotting (**h**–**l**). The number of apoptotic cells was determined by Annexin V-FITC and propidium iodide (PI) staining (**m**–**o**). **p**–**u** Cells were treated with apatinib (25 μM) with or without rapamycin (1 nM). The expression levels of related genes were measured by western blotting (**p**–**r**). The number of apoptotic cells was assessed by Annexin V-FITC and propidium iodide (PI) staining (s-u). * p < 0.05, ** p < 0.01, *** p < 0.001 compared with control group. ^#^ p < 0.05, ^##^ p < 0.01, ^###^ p < 0.001

Next, we further explored the specific mechanism by which apatinib affected autophagy and apoptosis in ESCC cells. It has been reported that ER stress was related to both autophagy and apoptosis [23], and the IRE-1α pathway was one of the three main pathways involved in ER stress. Many studies have shown that the AKT–mTOR pathway was closely related to autophagy and apoptosis [27, 28], but it remained unclear whether IRE-1α mediated the autophagy and/or apoptosis induced by apatinib through the AKT–mTOR pathway. IRE-1α siRNA was used to transfect ESCC cell lines. Figure 4a–g showed that knocking down IRE-1α inhibited the expression levels of ER stress- and autophagy-related proteins. In addition, knocking down IRE-1α also had a significant impact on apoptosis-related proteins (Fig. 4a–g). More interestingly, we found that after silencing IRE-1α, the activity levels of pAKT and pmTOR were also reduced, which suggested that IRE1-α might affect the regulation of autophagy and apoptosis by apatinib through the AKT–mTOR pathway (Fig. 4a–g). Then, the AKT activator SC79 was used alone or in combination with apatinib. The western blot results showed that compared with apatinib, the combination of the two drugs significantly inhibited the expression levels of Bax and LC3 II, but increased the expression levels of Bcl2, p62, pAKT and pmTOR which indicated that SC79 inhibited the activation of apatinib on autophagy and apoptosis (Fig. 4h–l). Flow cytometry was carried out to detect apoptotic cells. Figure 4m–o showed that compared with apatinib treatment alone, the combination of apatinib and SC79 significantly inhibited apoptosis. Rapamycin, an inhibitor of mTOR, was used to explore the influence of the mTOR pathway on the effect of apatinib. The western blot results showed that compared with apatinib treatment alone, the combination of apatinib and rapamycin inhibited the expression of Bcl2 but increased the expression levels of Bax and LC3 II (Fig. 4p–r). The results of the apoptosis detection assay by flow cytometry showed that compared with apatinib treatment alone, the combination of apatinib and rapamycin further increased the tumor cell apoptosis rate (Fig. 4s–u). These results indicated that the IRE-1α–AKT–mTOR pathway was involved in the regulation of autophagy and apoptosis by apatinib.

### Inhibiting autophagy increased apatinib-induced apoptosis

**Fig. 5:**
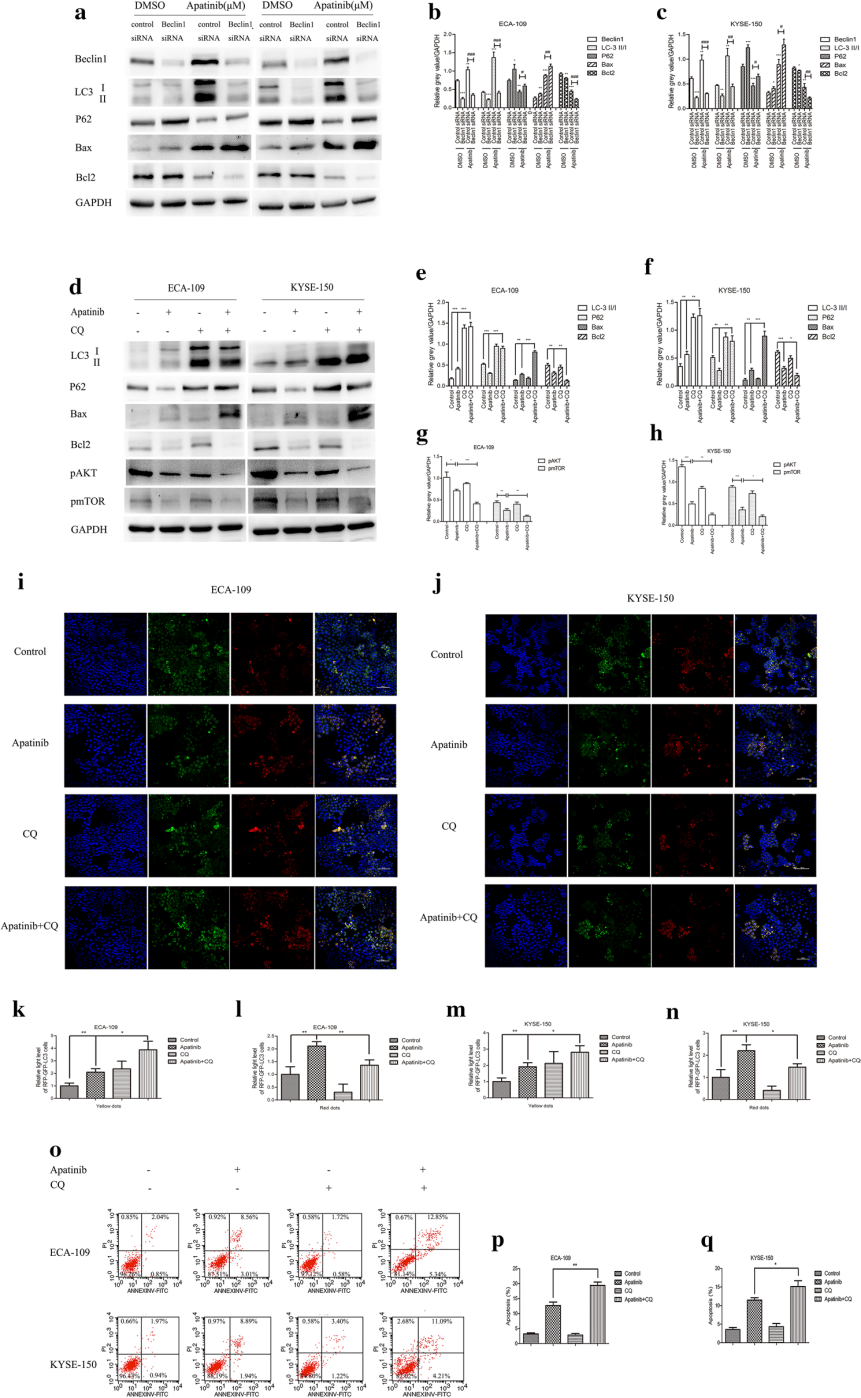
Inhibiting autophagy increased apatinib-induced apoptosis. **a**–**c** Cells were treated with 25 μM apatinib or DMSO for 24 h after transfection with control siRNA or Beclin1 siRNA. The expression levels of related genes were measured by western blotting. **d**–**q** Cells were pretreated with 10 μM CQ for 6 h and then treated with apatinib (25 μM) for 24 h. The expression levels of related genes were detected by western blotting (**d**–**h**). Representative images and quantification of autophagosomes shown as yellow dots and autolysosomes shown as red dots were presented (**i**–**n**). The number of apoptotic cells was assessed by Annexin V-FITC and propidium iodide (PI) staining (o-q). * p < 0.05, ** p < 0.01, *** p < 0.001 compared with control group. ^#^ p < 0.05, ^##^ p < 0.01, ^###^ p < 0.001

The aforementioned results indicated that apatinib regulated autophagy and apoptosis through the IRE-1α–AKT–mTOR pathway, but it remained unclear whether autophagy played a role in this process. First, Beclin1 siRNA was used to knock down Beclin1, and then, the effect of apatinib on the apoptosis of ESCC cells was determined. The western blot results showed that the expression of Bax was significantly increased after Beclin1 was knocked down, while the expression of Bcl2 was significantly decreased (Fig. 5a–c). CQ, an antimalarial lysosomal inhibitor, has been identified as an inhibitor of autophagy [29], and it has been found to prevent autophagy by blocking autophagosomal-lysosomal fusion [30]. The western blot results revealed that administration of CQ as a pretreatment led to significant accumulation of lipidated LC3 and P62, indicating that autophagy had been inhibited. Compared with apatinib treatment alone, the combination of CQ and apatinib significantly increased the expression of Bax and inhibited the expression levels of Bcl2, pAKT and pmTOR, which suggested that the inhibition of autophagy enhanced the effect of apatinib on tumor cell apoptosis (Fig. 5d–h). We used the RFP-GFP-LC3 lentivirus to transfect ESCC cells. The results of confocal microscopy indicated that compared with apatinib treatment alone, the combination of CQ and apatinib significantly increased the number of yellow spots, while the number of red spots decreased, indicating that the formation of autophagolysosomes was inhibited by CQ (Fig. 5i–n). Finally, flow cytometry was used to detect apoptotic cells. Figure 5o–q showed that the administration of CQ as a pretreatment significantly enhanced the induction of apoptosis by apatinib. In summary, we demonstrated that inhibition of autophagy enhanced the inducing effect of apatinib on tumor cell apoptosis.

### The combination of apatinib and CQ sensitized cells to paclitaxel to inhibit their proliferation and induce apoptosis

**Fig. 6:**
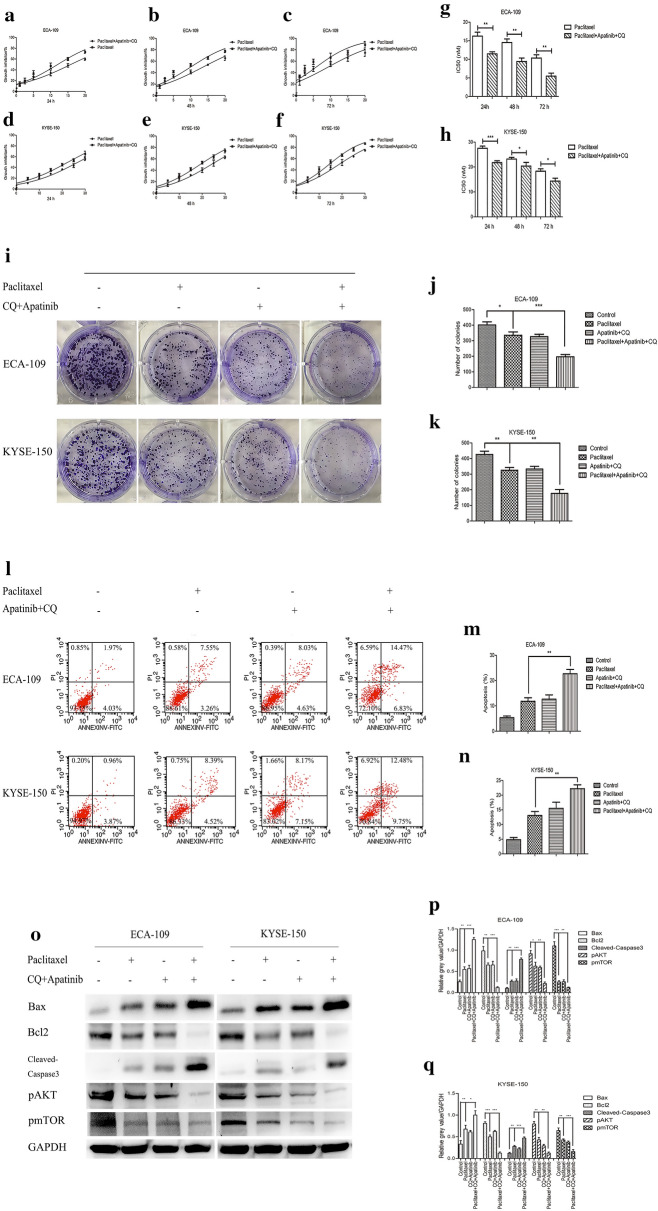
The combination of apatinib and CQ sensitized ESCC cells to paclitaxel, which inhibited their proliferation and induced apoptosis. **a**–**f** ECA-109 and KYSE-150 cells were treated with different concentrations of paclitaxel with or without apatinib (25 μM) and CQ (10 μM) for 24, 48 and 72 h and cell viability was evaluated by CCK-8 assay. **g**, **h** IC50 values were calculated. **i**–**k** Representative images of the colonies formed and colony quantification were shown. Cells were treated with paclitaxel (5 nM) with or without apatinib (25 μM) and CQ (10 μM). Apoptosis was assessed by Annexin V-FITC and propidium iodide (PI) staining (**l**–**n**). Western blotting was used to measure the expression levels of related genes (**o**–**q**). * p < 0.05, ** p < 0.01, *** p < 0.001 compared with control group

We further explored whether the combination of apatinib and CQ could sensitize cells to paclitaxel. Different concentrations of paclitaxel were used to treat ESCC cells with or without apatinib and CQ for 24, 48, and 72 h. The results indicated that apatinib and CQ significantly enhanced the inhibitory effect of paclitaxel on the proliferation of tumor cells and reduced the paclitaxel IC50 values (Fig. 6a–h). Plate clone formation experiments were used to further verify our conclusions. Compared with the effect of paclitaxel treatment alone, the combination of paclitaxel with apatinib and CQ significantly inhibited the formation of ESCC clones (Fig. 6i–k). Flow cytometry was used to evaluate the apoptosis rate of tumor cells. Figure 6l–n showed that apatinib and CQ significantly enhanced the rate of tumor cell apoptosis induced by paclitaxel. The western blot results revealed that when apatinib and CQ were combined with paclitaxel, the expression levels of Bax and Cleaved-Caspase3 were significantly increased, while the levels of Bcl2, pAKT and pmTOR were significantly reduced (Fig. 6o–q). These results suggested that apatinib and CQ could significantly enhance the inhibitory effect of paclitaxel on tumor cell proliferation and accelerate apoptosis.

### The IRE-1α–AKT–mTOR signaling pathway participated in the sensitization of cells to paclitaxel through the effects of apatinib and CQ

**Fig. 7:**
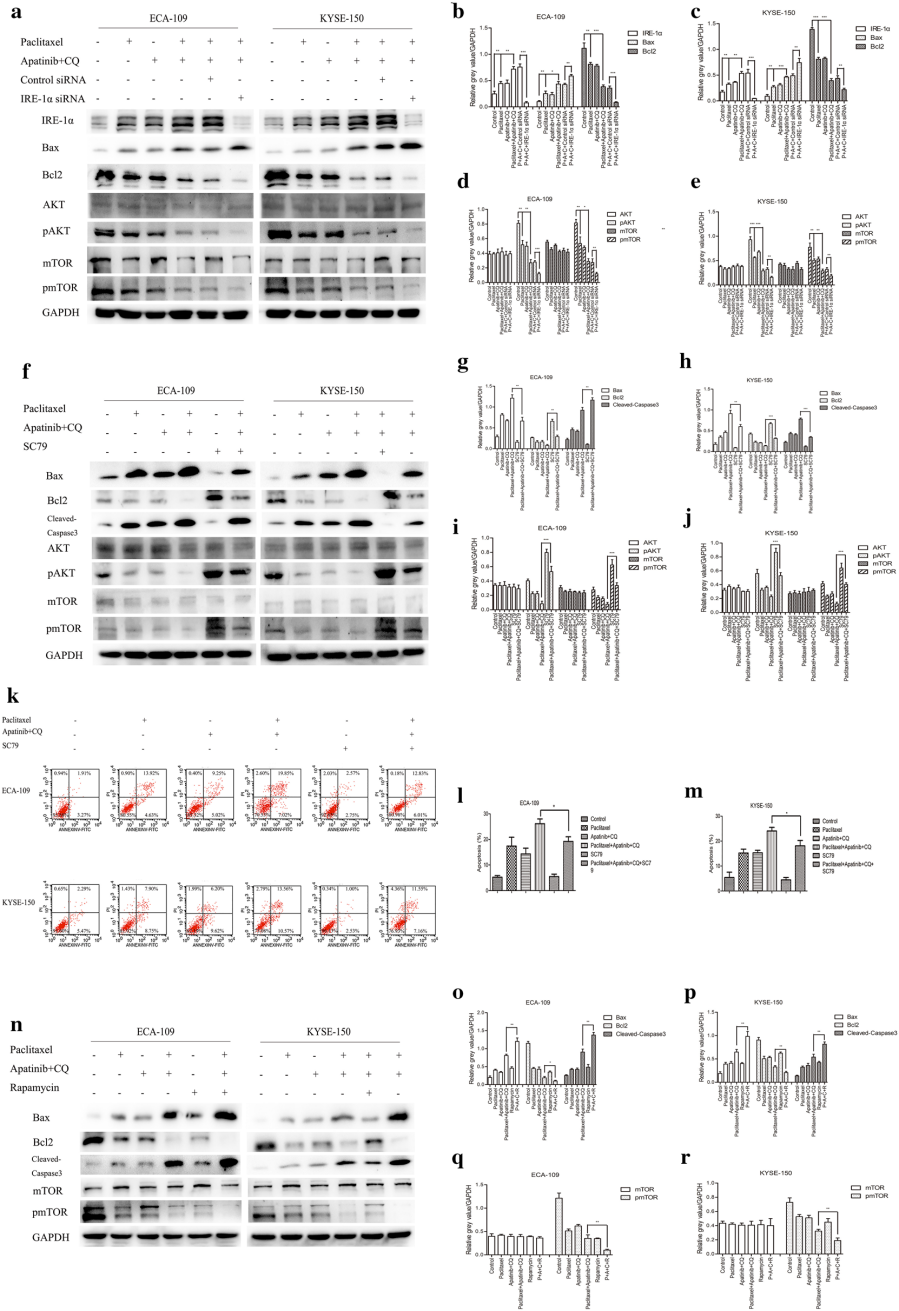
The combination of apatinib and CQ sensitized cells to paclitaxel, which induced their apoptosis via the IRE-1α–AKT–mTOR pathway. **a**–**e** ECA-109 and KYSE-150 cells were treated with 5 nM paclitaxel with or without apatinib and CQ for 24 h after transfection with control siRNA or IRE-1α siRNA. The expression levels of related genes were measured by western blotting. **f**–**m** Cells were treated with paclitaxel or apatinib and CQ with or without SC79 (4 μg/ml). The expression levels of related genes were measured by western blotting (**f**–**j**). The number of apoptotic cells was assessed by Annexin V-FITC and propidium iodide (PI) staining (**k**–**m**). **n**–**r** Cells were treated with paclitaxel or apatinib and CQ with or without rapamycin (1 nM). The expression levels of related genes were measured by western blotting. * p < 0.05, ** p < 0.01, *** p < 0.001 compared with control group. (P, paclitaxel; A, apatinib; C, CQ; R, rapamycin)

The results shown in Fig. 6o–q indicated that the combination of paclitaxel with apatinib and CQ further inhibited the expression levels of pAKT and pmTOR; however, it remained unknown whether the sensitization effect of apatinib and CQ on cell sensitization to paclitaxel mediated through the IRE-1α–AKT–mTOR pathway. IRE-1α siRNA was first transfected into ESCC cell lines, which were then treated with different drugs for 24 h after transfection. The western blot results showed that after knocking down IRE-1α, compared with the combination of paclitaxel with apatinib and CQ group, the expression level of Bax was further increased, and the expression levels of Bcl2, pAKT and pmTOR were further decreased in the IRE-1α-knockdown group (Fig. 7a–e). This result revealed that IRE-1α was involved in the sensitization of paclitaxel by apatinib and CQ, and was the upstream molecule of the AKT–mTOR pathway. SC79, an AKT activator, was used in combination with apatinib and CQ and paclitaxel or not. The western blot results showed that compared with the combination of paclitaxel with apatinib and CQ group, the addition of SC79 to this combination reduced the expression levels of Bax and Cleaved-Caspase3 and increased the expression levels of Bcl2, pAKT and pmTOR (Fig. 7f–j). The results of the apoptosis assay with flow cytometry verified that the addition of SC79 would reverse the inducing effect of paclitaxel combined with apatinib and CQ on the apoptosis of ESCC cells (Fig. 7k–m). Figure 7n–r showed the effect of the mTOR inhibitor rapamycin on the apoptosis induced by paclitaxel combined with apatinib and CQ. The western blot results showed that compared with paclitaxel combined with apatinib and CQ group, adding rapamycin to this combination further increased the expression levels of Bax and Cleaved-Caspase3, while the expression levels of Bcl2 and pmTOR were reduced. These results corroborated the findings showing that the facilitating effect of apatinib and CQ on cell sensitization to paclitaxel was regulated by the IRE-1α–AKT–mTOR pathway.

### The combination of apatinib and CQ intensified tumor suppression induced by paclitaxel

**Fig. 8:**
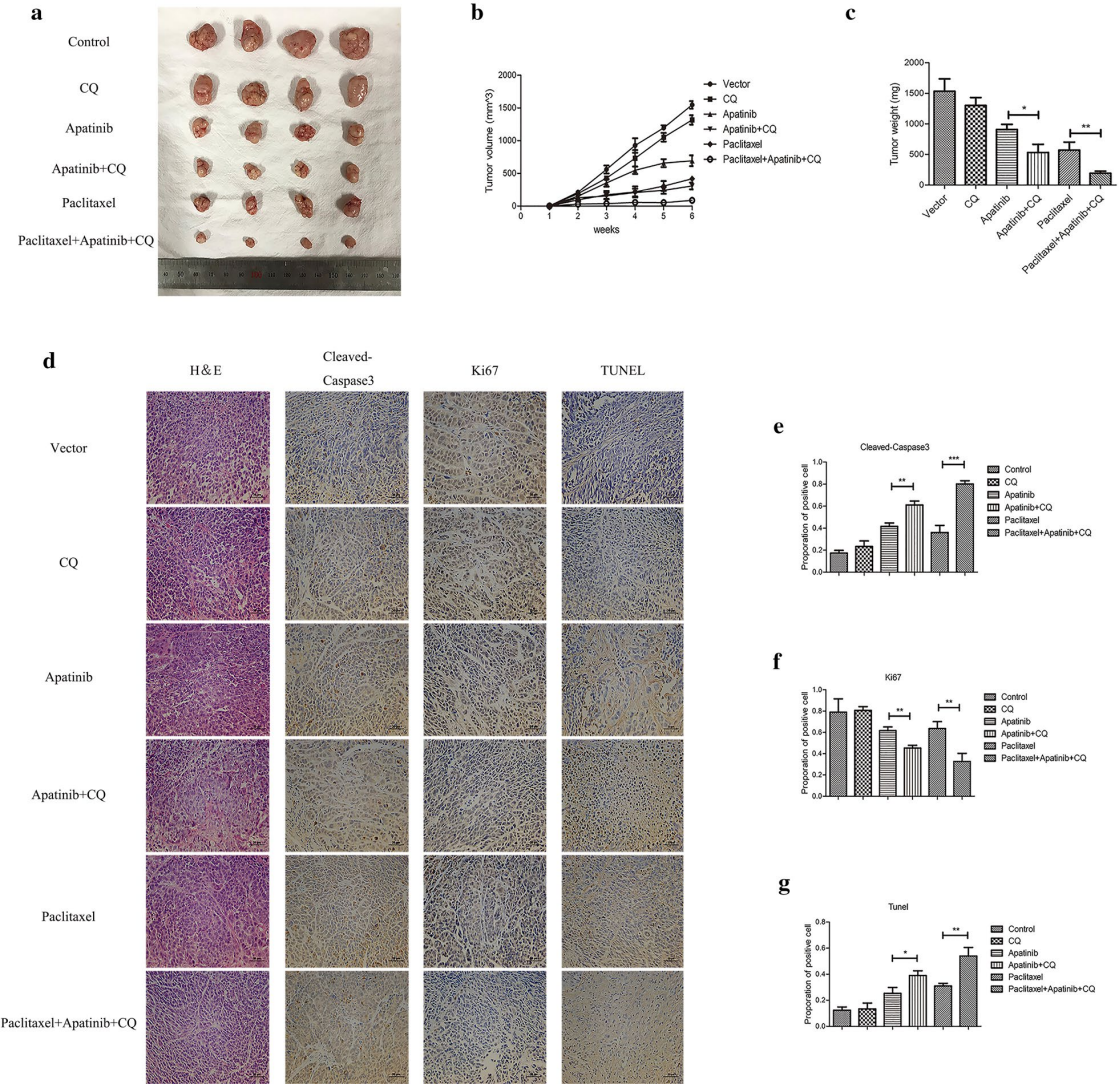
Inhibition of tumor growth in vivo by the combination of apatinib and CQ with paclitaxel. **a** Representative images of xenograft tumors were shown. **b** Tumor volume was assessed weekly. **c** Tumor weight was calculated for each group. **d** Representative IHC staining images of Cleaved-Caspase3, Ki67 were shown of every group and TUNEL assay was used for measuring apoptosis. **e**–**g** Quantitative analysis of IHC staining and TUNEL assay results. * p < 0.05, ** p < 0.01, *** p < 0.001 compared with control group

Considering the aforementioned results, we verified our conclusions through in vivo experiments. The ECA-109 cell line was used to inoculate nude mice subcutaneously. After two weeks, all the nude mice were assigned to 6 groups: control group, CQ group, apatinib group, apatinib and CQ group, paclitaxel group, paclitaxel combined with apatinib and CQ group. Four weeks after the drugs administration, all the mice were sacrificed, and the tumors were removed to measure the volume and weight. Figure 8a–c showed that compared with the effect of apatinib alone, the combination of CQ and apatinib significantly reduced tumor volume and weight (307.00 ± 42.01 mm^3^ (apatinib + CQ) vs 693.33 ± 67.99 mm^3^ (apatinib), p < 0.01; 533.33 ± 108.73 g (apatinib + CQ) vs. 906.67 ± 70.40 g (apatinib), p < 0.05). Compared with the control group mice, the paclitaxel group mice exhibited reduced tumor volume and weight to a certain extent (417.33 ± 24.78 mm^3^ (paclitaxel) vs 1547.33 ± 47.79 mm^3^ (control), p < 0.001; 570.00 ± 106.14 g (paclitaxel) vs 1533.33 ± 164.99 g (control), p < 0.01), and when paclitaxel was combined with apatinib and CQ, the reducing effects on tumor volume and weight were most obvious (86.83 ± 24.29 mm^3^ (paclitaxel + apatinib + CQ) vs 417.33 ± 24.78 mm^3^ (paclitaxel), p < 0.001; 192.33 ± 27.88 g (paclitaxel + apatinib + CQ) vs 570.00 ± 106.14 g (paclitaxel), p < 0.01). Immunohistochemistry was used to assess the expression of Ki67 and Cleaved-Caspase3 in different groups of tumor tissues, and TUNEL was used to determine the number of apoptotic cells in different groups of tumor tissues. Figure 8d–g showed that, compared with the apatinib group, the CQ and apatinib group showed more significantly inhibited Ki67 and Cleaved-Caspase3 expression and increased apoptosis in tumor tissue. Compared with the paclitaxel single-agent group, the paclitaxel combined with apatinib and CQ group significantly inhibited Ki67 expression and increased Cleaved- Caspase3 expression, and the apoptosis rate in tumor tissue was also increased significantly. These results corroborated the finding that apatinib and CQ improved the ability of paclitaxel to inhibit cell proliferation and promote apoptosis in ESCC tissues.

## Discussion

Esophageal squamous cell carcinoma is a major life-threatening malignancy characterized by high incidence and mortality, and early distant metastasis [31, 32]. Surgery plays a pivotal role in the treatment of T1 or T2 stage tumors, while neoadjuvant/adjuvant therapy is applied for locally advanced ESCC [33, 34]. Since ESCC is often detected at an advanced stage, chemotherapy and targeted therapy might be effective perioperative treatments [7]. At present, there are many types of drugs involved in targeted therapy of ESCC, but most researches about targeted therapy are still in the clinical or basic research stage and only 3 targeted therapeutic agents including trastuzumab, ramucirumab, and pembrolizumab, have been approved by the FDA for use in esophageal and EGJ cancers. In many studies, apatinib has been reported not only to exert antitumor effects but also to enhance the sensitivity of tumor cells to chemotherapeutic agents. Huang et al. reported that apatinib inhibited the proliferation, migration and invasion of cholangiocarcinoma cells [35]. Meng et al. found that apatinib inhibited cell proliferation and induced autophagy in human papillary thyroid carcinoma [36]. Xu et al. proved that apatinib enhanced the chemosensitivity of gastric cancer cells to paclitaxel and 5-fluorouracil [37]. Moreover, Li et al. found that apatinib notably enhanced the antitumor activity of gefitinib in NSCLC cells and in xenografts carrying T790M mutations [38]. So far, there have been few studies on apatinib application in the treatment of ESCC. Wei et al. reported that apatinib suppressed tumor progression and enhanced cisplatin sensitivity in esophageal cancer via the Akt/β‑catenin pathway [18]. The role of apatinib in ESCC and related mechanisms still need to be further clarified. And whether apatinib had a sensitizing effect on paclitaxel and the underlying mechanism in ESCC remained to be explored. In our study, we first clarified the inhibitory effects of apatinib on the cell proliferation, invasion, and migration of ESCC cell lines. Then, we found that apatinib induced ER stress, autophagy and apoptosis. Inhibiting autophagy could enhance apatinib-induced apoptosis. Finally, we proved that the combined application of apatinib and CQ sensitized cells to paclitaxel, which induced apoptosis through the IRE-1α–AKT–mTOR signaling pathway.

It has been reported that there was a close relationship among ER stress, autophagy and apoptosis [39–41]. Generally, autophagy is activated under ER stress. Autophagy induced by ER stress plays both survival- and death-promoting roles. Most relevant studies have demonstrated that autophagy was activated to inhibit apoptosis after ER stress [42, 43]. We aimed to clarify the relationship among ER stress, autophagy and apoptosis, because it was essential for the treatment of ESCC. In our study, we found that apatinib induced ER stress, autophagy and apoptosis of ESCC cells. Knockdown of IRE-1α, which was the key gene in one of three major ER stress signaling pathways, inhibited apatinib-induced autophagy and enhanced apatinib-induced apoptosis. This result indicated that ER stress was the upstream mechanism of apatinib-induced autophagy and apoptosis. Moreover, we found that knocking down IRE-1α enhanced the inhibitory effects of apatinib on pAKT and pmTOR expression levels. The AKT–mTOR signaling pathway has been reported to be closely related to tumor survival [27, 28]. Thus, SC79 (an AKT activator) and Rapamycin (an mTOR inhibitor) were used to clarify the role of the AKT–mTOR signaling pathway in apatinib-induced autophagy and apoptosis. The results showed that inhibiting the AKT–mTOR pathway increased the expression levels of autophagy- and apoptosis-related proteins and was the downstream mechanism of IRE-1α. Beclin1-siRNA and CQ, which were autophagy inhibitors, were used to clarify the relationship between autophagy and apoptosis. The results showed that inhibiting autophagy by CQ or Beclin1-siRNA promoted the apoptosis of ESCC cells induced by apatinib. Through this part of the study, we confirmed that apatinib could activate ER stress, autophagy, and apoptosis. And the activating effect of apatinib on autophagy and apoptosis was dependent on endoplasmic reticulum stress. Furthermore, inhibiting autophagy increased apatinib-induced apoptosis.

Monotherapy with apatinib appeared to be effective only for selected tumors that were highly dependent on VEGF. Relevant studies have proved that combination of anti-angiogenic targeted therapy and conventional chemotherapy might lead to improved response rates in several solid tumors rates, including ovarian cancer, breast cancer and colon cancer [37]. Considering the synergistic effect of apatinib and CQ, we further explored whether the two drugs sensitized cells to paclitaxel. In our study, we demonstrated that the combination of apatinib and CQ enhanced the inhibition of paclitaxel on the proliferation of ESCC cells and promoted apoptosis. The aforementioned results indicated that apatinib regulated autophagy and apoptosis through the IRE-1α–AKT–mTOR pathway. We wanted to further elucidate the mechanism by which apatinib and CQ sensitized cells to paclitaxel. IRE-1α siRNA was used to knock down IRE-1α. SC79 was used to activate expression levels of pAKT and rapamycin was used to inhibit expression levels of pmTOR. The results showed that the combination of apatinib and CQ sensitized ESCC cells to paclitaxel to induce apoptosis through the IRE-1α–AKT–mTOR signaling pathway both in vivo and in vitro.

In this study, we discovered that apatinib induced ER stress, autophagy and apoptosis in ESCC. Inhibiting autophagy by CQ enhanced apatinib-induced apoptosis. The combination of apatinib and CQ sensitized ESCC cells to paclitaxel to induce apoptosis through the IRE-1α–AKT–mTOR signaling pathway. Our study has potential clinical application value and may provide new ideas for the comprehensive diagnosis and treatment of esophageal cancer.

## Supplementary Information

**Additional file 1: Figure S1.** Construction of IRE-1α and Beclin 1 gene knock-down cell models. (a-c) ECA-109 and KYSE-150 cells were transfected with NC-siRNA or IRE-1α-siRNAs for 24 h. The expression level of IRE-1α was detected by Western blot. (d-f) ECA-109 and KYSE-150 cells were transfected with NC-siRNA or Beclin 1-siRNAs for 24 h. The expression level of Beclin 1 was detected by Western blot.

## Data Availability

All data used and/or analyzed in the current study are available from the corresponding author on reasonable request.
